# Bistable fully developed mixed convection flow with viscous dissipation in a vertical channel

**DOI:** 10.1098/rsos.171880

**Published:** 2018-07-11

**Authors:** M. Miklavčič

**Affiliations:** Department of Mathematics, Michigan State University, East Lansing, MI 48824, USA

**Keywords:** viscous, convection, stability, dual solutions, thermal radiation, hysteresis

## Abstract

It is shown that unstable dual solutions in fully developed mixed convection flow in a vertical channel disappear in the presence of relatively strong thermal radiation. In this case, we have a unique stable flow at each pressure gradient. When the effect of thermal radiation is weak another branch of stable solutions is created, resulting in bistable flows. In this case, the flow exhibits hysteresis with variation of the pressure gradient. Optically, a thin radiation model is used.

## 1. Introduction

Dual mixed convection flows have been studied for a long time. Joseph [1,2] studied them for Couette and Poiseuille flows. Wilks & Barmley [3] observed them in boundary layers. In these cases, they established instability of one branch of flows and existence of the critical stress. Barletta *et al.* [4] found dual solutions in vertical channels and the existence of a critical stress (minimum pressure gradient). Instability of one branch of flows in vertical channels was established by Miklavčič [5] and by Barletta & Miklavčič [6]. These unstable dual solutions are due to the nonlinear viscous term in the energy balance equation and are discussed in detail by Barletta [7]. Here, the focus is on the effects of thermal radiation on such flows. It is shown that the dual solutions in vertical channels disappear when the effect of thermal radiation is strong enough. In this case, one unique stable flow appears at all pressure gradients and there is no critical stress. On the other hand, when thermal radiation is relatively weak, another stable branch of flows appears in addition to the unstable dual branch. This creates bistable flows and the effect of the pressure gradient on the flow exhibits hysteresis. An evaluation of parameters in the cases of water and air flows shows that both scenarios are within the realm of possibility. On the new branch of solutions, the temperature attains the maximum near the walls. Laminar flows with hysteresis are rather rare but have been observed before; see, for example, [8].

## 2. Model

We consider a fully developed viscous flow in a vertical channel between parallel plates −*L* < y* < *L* with wall temperature *T*_a_. Gravitational acceleration points in the direction of the negative *z*-axis. The flow is driven by a constant vertical pressure gradient ∂*p*/∂z* and by the buoyancy force induced by the temperature gradient. In a fully developed regime, the velocity field is parallel to the *z*-axis and expressed by its *z*-component W*. Using Oberbeck–Boussinesq approximation [5,9,10], the local momentum balance equation and the local energy balance equations can be formulated as

2.1 $$\begin{array}{rl} \rho \frac{\partial W^{*}}{\partial t^{*}} & =\mu \frac{\partial^{2}W^{*}}{\partial y^{*2}}-\frac{\partial p}{\partial z^{*}}+\rho g\beta (T-T_{a}), \end{array}$$

2.2 $$\begin{array}{rl} \rho c\frac{\partial T}{\partial t^{*}} & =k\frac{\partial^{2}T}{\partial y^{*2}}+\mu {\left(\frac{\partial W^{*}}{\partial y^{*}}\right)}^{2}-\frac{\sigma (T^{4}-T_{a}^{4})}{2L} \end{array}$$

2.3 $$\begin{array}{rl} \;\text{and}\;W^{*} & =0,\;T=T_{a}\;\text{at }y^{*}=\pm L. \end{array}$$

Here, *ρ* denotes the density, *μ* the dynamic viscosity, *β* is the coefficient of thermal expansion, *k* is the thermal conductivity and *c* is the specific heat of the fluid. *g* is the gravitational acceleration and *σ* is the Stefan–Boltzmann constant.

Equation (2.1) is often stated [6,10,11] with a mean temperature

$$T_{m}=\frac{1}{2L}\int_{-L}^{L}T\;dy^{*},$$

in place of the fixed ambient temperature *T*_a_. Different choices produce different quantative results, but the qualitative behaviour remains the same [5,6]. The nonlinear optically thin thermal radiation approximation *σ*(*T*^4^ − *T*^4^_a_) in equation (2.2) has been used before when studying the heat transfer in a medium between parallel walls [12–14]. It is divided by 2*L* because the energy balance equation (2.2) is given per unit volume. In applications, one should consider replacing the Stefan–Boltzmann constant *σ* with one that more properly reflects absorption and reflectivity.

Using scaling

2.4 $$y=\frac{y*}{L},\;t=t^{*}\frac{\mu }{L^{2}\rho },\;w=W^{*}\frac{\rho g\beta L^{2}}{k},\;\theta =(T-T_{a})\frac{{\left(\rho g\beta L^{2}\right)}^{2}}{k\mu }$$

in equations (2.1)–(2.3) gives

2.5 $$\begin{array}{rl} \frac{\partial w}{\partial t} & =\frac{\partial^{2}w}{\partial y^{2}}-\Pi +\theta , \end{array}$$

2.6 $$\begin{array}{rl} Pr\frac{\partial \theta }{\partial t} & =\frac{\partial^{2}\theta }{\partial y^{2}}+{\left(\frac{\partial w}{\partial y}\right)}^{2}-\varepsilon \theta (4+6r\theta +4r^{2}\theta^{2}+r^{3}\theta^{3}) \end{array}$$

2.7 $$\begin{array}{rl} \;\text{and}\;w & =0,\;\theta =0\;\text{at }y=\pm 1, \end{array}$$

where

2.8 $$Pr=\frac{c\mu }{k},\;\varepsilon =\frac{LT_{a}^{3}\sigma }{2k},\;r=\frac{k\mu }{{\left(\rho g\beta L^{2}\right)}^{2}T_{a}},\;\Pi =\frac{\rho g\beta L^{4}}{k\mu }\frac{\partial p}{\partial z^{*}}.$$

*Pr* is the Prandtl number. The values *ε* and *r* greatly depend on *L*. Some sample values of the parameters are listed in table 1.

**Table 1.** Using data at 300 K, normal atmospheric pressure and channel width 2*L* = 0.1 m.

	*Pr*	*ε*	*r*
air	0.69	1.5	0.16
water	6.9	0.065	0.073

## 3. Stationary flows

The time-independent solutions of equations (2.5) and (2.6) satisfy

3.1 $$0=w^{\prime \prime }-\Pi +\theta$$

and

3.2 $$0=\theta^{\prime \prime }+{\left(w^{\prime }\right)}^{2}-\varepsilon \theta (4+6r\theta +4r^{2}\theta^{2}+r^{3}\theta^{3}),$$

on the interval ( − 1, 1) and boundary conditions given by (2.7). Symmetry implies

3.3 $$w^{\prime }(0)=\theta^{\prime }(0)=0\;\mathrm{and}\;w(1)=\theta (1)=0.$$

For any given values of *Π*, *w*(0), *θ*(0), we get at most one solution of (3.1)–(3.3). We pick one of the three and use the two-dimensional Newton's method to find the other two values so that the two conditions *w*(1) = *θ*(1) = 0 are met. Some sample values are listed in table 2.

**Table 2.** Data for the stationary flows marked in figures 1, 3, 4 when *ε* = 0.01 and *r* = 0.1.

flow	*Π*	*w*(0)	*θ*(0)	λ_1_
A	12	-3.834986	4.608805	−1.86858 ± 1.89609i
B	0	0	0	−(16*ε* + *π*^2^)/(4*Pr*)
C	-3.221423	3.219782	3.443091	0
D	0	6.688761	14.207656	0.60146
E	13.764045	20.771750	50.348734	0
F	0	42.864480	66.340718	−1.04022

**Figure 1.**
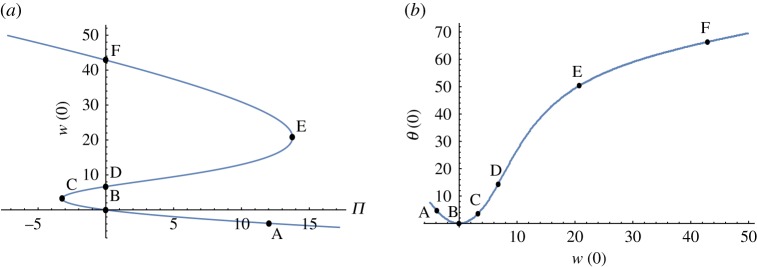
Stationary flows when *ε* = 0.01 and *r* = 0.1.

A good overview of stationary flows is shown in figure 1 for the case when *ε* = 0.01 and *r* = 0.1. Using data for water in table 1, we get similar curves.

**Figure 2.**
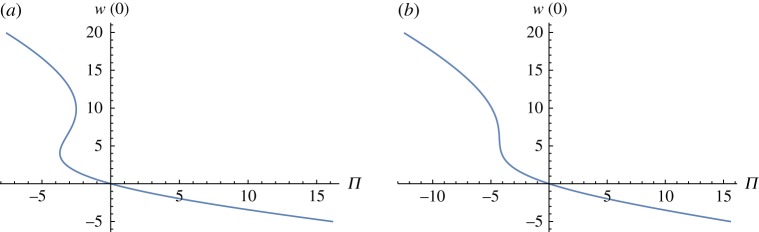
Stationary flows when *r* = 0.1 and *ε* increases from 0.06 (*a*) to 0.1 (*b*).

If *ε* (or *r*) is increased, the second turning point E moves to the left and it disappears as shown in figure 2. Using data for air in table 1, we get a curve that is a bit more stretched out than the curve in figure 2*b*.

If *ε* (or *r*) is decreased, the second turning point E moves to the right towards infinity. When *ε* = 0, the no radiation case, there is no second turning point and no third branch and we are left with the mixed convection flows studied before [5,6]. As *ε* → 0, the flow marked by D on figure 1*a*,*b* approaches the flow called completely passive natural convection flow with *w*(0) = 6.111 and *θ*(0) = 13.155 [15]—which are not too far from the values given in table 2 for D. However, the transition *ε* → 0 is rather complicated far from the origin as the radiation term is a singular perturbation at high temperatures.

**Figure 3.**
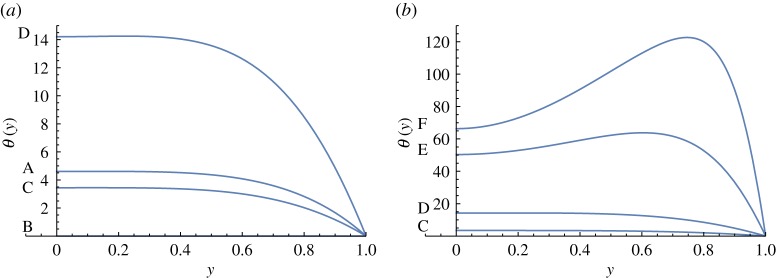
Temperature distributions when *ε* = 0.01 and *r* = 0.1.

The rest state is at point B in figure 1 where *Π* = *w* = *θ* = 0. Along the path from B to F, the temperature monotonically increases, as shown in figure 3. However, the temperature increases also as we move on the lower branch towards A. Observe that the temperature is much higher near the wall at F.

**Figure 4.**
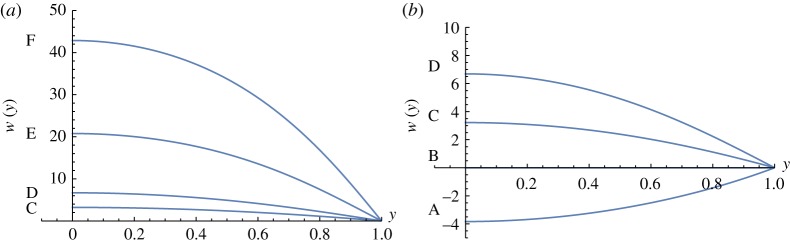
Velocities when *ε* = 0.01 and *r* = 0.1.

Figure 4 shows that velocities monotonically increase all the way from A to F. In the no radiation case, back flows were found [5,6]; however, in the region of the values of parameters considered here, there are no back flows. When moving from B towards F in figure 1, velocity profiles start having inflection points at D, just as in the no radiation case, even though this is not obvious in figure 4.

If one were to use *T*_m_ in place of *T*_a_ in equation (2.1), then the stationary solutions would be exactly the same provided that the pressure gradient is shifted as follows:

$$\Pi_{\mathrm{using}\;T_{m}}=\Pi -\int_{0}^{1}\theta \;dy.$$

## 4. Stability

Let *w*, *θ* be even stationary solutions of (3.1)–(3.3) at some *Π*. If perturbations

$$w(y)+u_{1}(y,t)\;\mathrm{and}\;\theta (y)+u_{2}(y,t)$$

satisfy equations (2.5)–(2.7), then

4.1 $$\begin{array}{rl} \frac{\partial u_{1}}{\partial t} & =\frac{\partial^{2}u_{1}}{\partial y^{2}}+u_{2} \end{array}$$

4.2 $$\begin{array}{rl} Pr\frac{\partial u_{2}}{\partial t} & =\frac{\partial^{2}u_{2}}{\partial y^{2}}+2w^{\prime }\frac{\partial u_{1}}{\partial y}+{\left(\frac{\partial u_{1}}{\partial y}\right)}^{2}-\varepsilon u_{2}(4{\left(1+r\theta \right)}^{3}+6{\left(1+r\theta \right)}^{2}ru_{2}+4(1+r\theta )r^{2}u_{2}^{2}+r^{3}u_{2}^{3}) \end{array}$$

4.3 $$\begin{array}{rl} \;\text{and}\;u_{1} & =0,\;u_{2}=0\;\text{at }y=\pm 1. \end{array}$$

This system can be considered as an abstract evolution equation

4.4 $$\frac{\partial u}{\partial t}+Au=f(u),$$

of *u* = (*u*_1_, *u*_2_) in (*L*^2^( − 1, 1))^2^, where the linear operator *A* is given by

4.5 $${\left(Au\right)}_{1}=-u_{1}^{\prime \prime }-u_{2},\;{\left(Au\right)}_{2}=\frac{-u_{2}^{\prime \prime }-2w^{\prime }u_{1}^{\prime }+4\varepsilon {\left(1+r\theta \right)}^{3}u_{2}}{Pr},$$

with the domain *D*(*A*) = (*W*^1^_0_( − 1, 1)∩*W*^2^( − 1, 1))^2^ and

4.6 $$f{\left(u\right)}_{1}=0,\;f{\left(u\right)}_{2}=\frac{{\left(u_{1}^{\prime }\right)}^{2}-\varepsilon ru_{2}^{2}(6{\left(1+r\theta \right)}^{2}+4(1+r\theta )ru_{2}+r^{2}u_{2}^{2})}{Pr}.$$

One can prove that (4.4) is a semilinear parabolic equation [16,17] and that *A* has compact resolvent. Hence, the well-known stability theory for semilinear parabolic equations (see, for example, [17]) can be applied to show that (nonlinear) stability of stationary solutions *w*, *θ* is determined by the eigenvalues of *A*. −λ is an eigenvalue of *A* if there exists a non-trivial solution of

4.7 $$\begin{array}{rl} & \lambda v=v^{\prime \prime }+\tau , \end{array}$$

4.8 $$\begin{array}{rl} & Pr\;\lambda \tau =\tau^{\prime \prime }+2w^{\prime }v^{\prime }-4\varepsilon {\left(1+r\theta \right)}^{3}\tau \end{array}$$

4.9 $$\begin{array}{rl} \;\text{and}\; & v=\tau =0\;\text{at }y=\pm 1. \end{array}$$

We will denote by λ_1_ the eigenvalue with the largest real part. If Re(λ_1_) < 0, then all solutions of the nonlinear equation (4.4) that are initially small enough will decay (p. 265 in [17]). Hence, all small perturbations will decay if Re(λ_1_) < 0. If Re(λ_1_) > 0, then one can find arbitrarily small perturbations of the stationary solution which evolve according to the nonlinear equation (4.4) to eventually become larger than a fixed threshold (p. 266 in [17]).

The central difference approximations were used to discretize (4.7) and (4.8) and then the eigenvalues of the corresponding matrix were calculated. Richardson extrapolation was used to improve accuracy. For reference values, see table 2. For the rest state *w* = *θ* = *Π* = 0, the eigenvalues are equal to

4.10 $$-\frac{{\left(n\pi \right)}^{2}}{4}\;\mathrm{and}\;-\frac{16\varepsilon +{\left(n\pi \right)}^{2}}{4Pr},\;\text{where }n=1,2,\ldots ,$$

hence the rest state is always stable.

**Figure 5.**
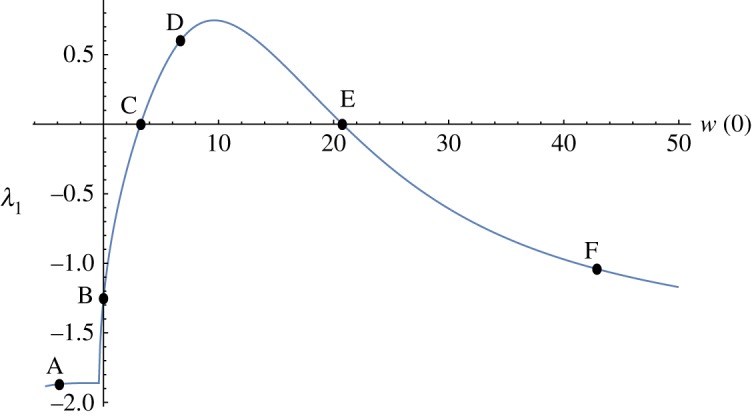
The leading eigenvalue, when *Pr* = 2, *ε* = 0.01 and *r* = 0.1.

Figure 5 shows the leading eigenvalue of stationary flows mentioned above. Observe that λ_1_ > 0 between C and E, hence the flows on the middle branch in figure 1 are unstable and the flows on the upper and the lower branches in figure 1 are stable.

The change of stability occurs at exactly the turning points C and E, as the following argument proves. Consider the solutions *w* and *θ* of (3.1)–(3.3) as functions of *ξ* = *w*(0) and define

$$v=\frac{\partial w}{\partial \xi }\;\mathrm{and}\;\tau =\frac{\partial \theta }{\partial \xi }.$$

If we differentiate (3.1)–(3.3) with respect to *ξ* and keep using prime to denote ∂/∂*y*, we obtain

4.11 $$\begin{array}{rl} & \frac{\partial \Pi }{\partial \xi }=v^{\prime \prime }+\tau , \end{array}$$

4.12 $$\begin{array}{rl} & 0=\tau^{\prime \prime }+2w^{\prime }v^{\prime }-4\varepsilon {\left(1+r\theta \right)}^{3}\tau \end{array}$$

4.13 $$\begin{array}{rl} \;\text{and}\; & v^{\prime }=\tau^{\prime }=0\;\text{at }y=0,\;v=\tau =0\;\text{at }y=1,\;v(0)=1. \end{array}$$

At the turning points ∂*Π*/∂*ξ* = 0; hence, at the turning points (4.7)–(4.9) have a non-trivial solution, with λ = 0. This shows an existence of eigenvalue λ = 0 at the turning points and, in the range of parameters under consideration, this happens to be the leading eigenvalue.

As the branch between C and E in figure 1 consists of unstable stationary solutions, flow velocities and temperatures exhibit hysteresis with variation of the pressure gradient.

**Figure 6.**
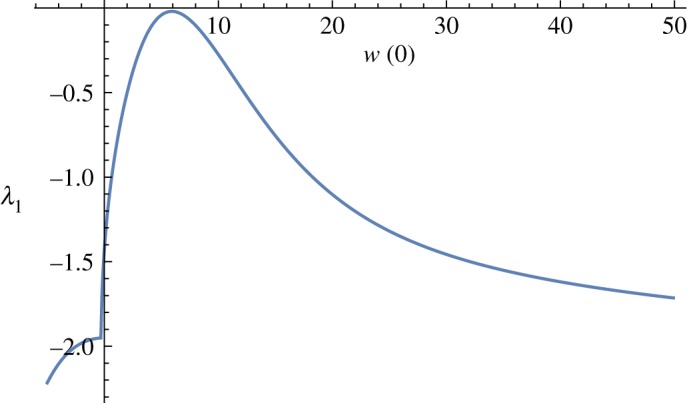
The leading eigenvalue, when *Pr* = 2, *ε* = 0.1 and *r* = 0.1.

If *ε* (or *r*) is increased, the unstable bump in figure 5 moves down until it sinks into the negative half-plane near *ε* = 0.1 (figure 6), indicating stability of all solutions.

If *ε* (or *r*) is decreased, the unstable bump on figure 5 moves up. When *ε* = 0, the leading eigenvalue simply increases after the turning point [5,6]. Introduction of radiation *ε* > 0 has a stabilizing effect, which is rather obvious in view of equation (2.6).

## 5. Direct evolution calculation

Starting with any initial velocity and temperature distributions, one would expect that evolution equations (2.5)–(2.7) would make those distributions approach the nearest stable stationary solution. To illustrate this, we chose initial distributions to be

5.1 $$w(0,y)=0,\;\theta (0,y)=(1-y^{2})\theta_{\max}\;\text{for }-1\leq y\leq 1,$$

i.e. we start with a stationary heated fluid, and we let *w* and *θ* evolve according to equations (2.5)–(2.7)—with no imposed pressure gradient (*Π* = 0). Results are presented for the case when *ε* = 0.01, *r* = 0.1 (effect of thermal radiation is weak) and *Pr* = 2.

When a stationary fluid, under no pressure gradient, is initially heated slightly, buoyancy starts the fluid motion, but then the fluid returns to the resting state. This is a normal expected outcome and it always happens when the effect of thermal radiation is strong enough. However, when *ε* = 0.01, *r* = 0.1 the effect of thermal radiation is weak, and when the maximum initial temperature $\theta_{\max}$ is sufficiently large, the fluid state approaches the stable equilibrium F on the upper branch in figure 1. During the approach to the stable equilibrium F, the fluid sustains high temperatures near the wall.

**Figure 7.**
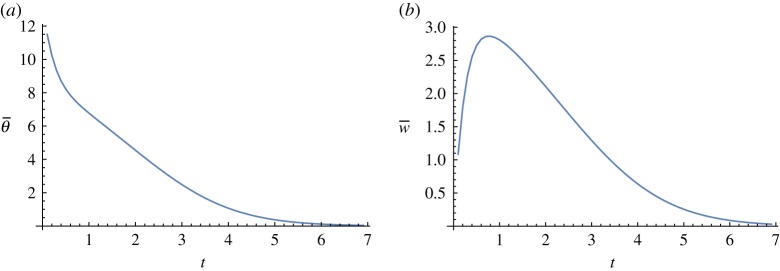
(*a*) Average temperature $\left(\bar{\theta }\right)$ and (*b*) average velocity $\left(\bar{w}\right)$ when $\theta_{\max}=20$ in (5.1).

For values of $\theta_{\max}\leq 28$, the solution approaches the rest state B. The average temperature simply decays to 0. The average velocity starts at zero, peaks and then decays to 0. A typical example can be seen in figure 7. The decay is exponential for both, and the decay rate approaches the first eigenvalue as time increases. The observed decay rate of the average temperature near *t* = 10 is −1.2532, while the first eigenvalue is −1.2537. Note also that the solution must pass close to the unstable stationary flow D.

**Figure 8.**
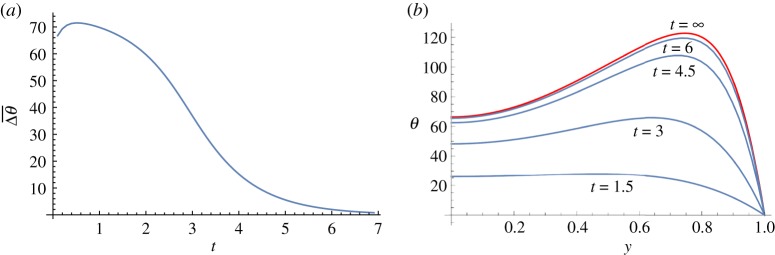
Approach to the stable solution F in figure 1 when $\theta_{\max}=40$ in (5.1). (*a*) The average difference between the temperature at time *t* and the limiting temperature. (*b*) The temperature distributions at times 1.5, 3, 4.5, 6 and ∞—the limiting one.

When $\theta_{\max}\geq 29$, the solution approaches the stable solution on the upper branch. An example is shown in figure 8. Note that on the upper branch the temperature in the middle of the channel is 66, and its maximum near the wall is about 123, yet in its basin of attraction is the state with temperature distribution that has maximum 29 in the centre—and 0 velocity. Hence, it has a ‘large’ basin of attraction. The decay of the average difference between the solution and the limiting solution is exponential, and the decay rate of the average temperature difference near *t* = 10 is −1.0396, which matches well with the first eigenvalue λ_1_ = − 1.0402.

## 6. Conclusion

A new kind of stable convection flows of a viscous fluid are shown to exist when the effect of thermal radiation is weak. They attract flows that are initially sufficiently hot. The maximum temperature of the new flows is attained near the walls, which may trigger interaction with the wall. Flows exhibit hysteresis with variation of the pressure gradient.

When the effect of thermal radiation is strong enough, the unstable dual branch disappears. In this case, we get a unique flow at each pressure gradient and there is no critical stress.

## Data accessibility

This work does not have any experimental data.
